# Use of a Bioresorbable Antibacterial Coating (DAC^®^ Hydrogel) in One-Stage Revision of Infected Knee Arthroplasty: Long-Term Study

**DOI:** 10.3390/antibiotics15080791

**Published:** 2026-08-15

**Authors:** Nicola Logoluso, Antonio Pellegrini, Delia Romano’, Valerio Pascale, Virginia Suardi, Carlo Luca Romano’

**Affiliations:** 1 I.R.C.C.S. Istituto Ortopedico Galeazzi, Centro di Chirurgia Ricostruttiva e delle Infezioni Osteoarticolari, Via Cristina Belgioioso 173, 20157 Milano, Italy; nicola.logoluso@grupposandonato.it (N.L.); delia-romano@libero.it (D.R.); 2 ASST Centro Specialistico Ortopedico Traumatologico Gaetano Pini-CTO, U.O.C Chirurgia Ricostruttiva delle Infezioni, Piazza Cardinal Ferrari 1, 20122 Milano, Italy; antonio.pellegrini@asst-pini-cto.it (A.P.); virginia.suardi@asst-pini-cto.it (V.S.); 3 I.R.C.C.S. Istituto Ortopedico Galeazzi, Ortopedia Clinicizzata, Via Cristina Belgioioso 173, 20157 Milano, Italy; valerio.pascale@grupposandonato.it; 4 Dipartimento di Scienze Biomediche per la Salute, Università degli Studi di Milano, 20122 Milano, Italy; 5 Romano Institute, Rruga Kongresi i Manastirit, Kompleksi Zirkon, 1007 Tirana, Albania

**Keywords:** knee, peri-prosthetic joint infection, PJI, one-stage revision, DAC, hydrogel, coating

## Abstract

Background/Objectives: One-stage revision has become an increasingly accepted strategy for the treatment of chronic periprosthetic joint infection (PJI) of the knee in carefully selected patients. The use of a resorbable defensive antibacterial coating (DAC^®^ hydrogel) represents an attractive adjunct to reduce bacterial adhesion and biofilm formation on revision implants. However, evidence regarding its long-term performance is limited. Methods: We retrospectively reviewed 52 consecutive patients undergoing one-stage revision for knee PJI or an infected spacer between 2014 and 2023 using a standardized surgical and antimicrobial protocol including local application of DAC hydrogel loaded with vancomycin alone or vancomycin plus meropenem. Clinical, microbiological, and radiographic outcomes were evaluated. Infection-free survival was assessed using Kaplan–Meier analysis. Results: Forty-eight patients underwent revision for PJI and four for septic spacers. Four patients were unavailable for final evaluation. At follow-up of 7.7 ± 2.9 years (range 2.5–12.2 years), 47 of 48 clinically evaluable patients remained infection-free, corresponding to a crude infection-free proportion of 97.9%. Kaplan–Meier infection-free survivorship at 12 years was 98.1% (95% CI, 87.1–99.7%). Mean Knee Society Score improved from 47.7 to 84.4 points, while functional KSS improved from 41.9 to 82.9 points. Pain at rest decreased from 6.9 to 0.7 and pain during movement from 8.1 to 1.6 on the visual analogue scale. Conclusions: This series represents, to our knowledge, the longest reported follow-up of one-stage knee revision using the DAC hydrogel coating. The favorable outcomes observed with this hybrid fixation strategy support further investigation of this approach in larger, long-term, comparative multicenter studies.

## 1. Introduction

Total knee arthroplasty (TKA) is among the most successful procedures in orthopedic surgery, providing reliable pain relief, restoration of function, and substantial improvement in quality of life for patients with end-stage knee disease. Owing to an aging population, increasing life expectancy, and expanding indications, the annual number of primary TKAs continues to rise worldwide. It has been estimated that more than 3.4 million primary TKAs will be performed annually in the United States by 2030, representing an increase of more than six-fold compared with 2005 [1]. Despite these excellent outcomes, periprosthetic joint infection (PJI) remains one of the most devastating complications following arthroplasty, accounting for a large proportion of early revision procedures and imposing a considerable economic burden on healthcare systems [2]. It has been estimated that, by 2030, the annual cost of treating PJI in TKA in the United States will be US$1.8 billion [3].

For decades, two-stage exchange arthroplasty has been regarded as the reference treatment for chronic knee PJI (defined as persistence of symptoms of infection for more than 4 weeks), particularly in the United States of America [4]. However, growing evidence accumulated during the last two decades has progressively challenged this paradigm. Several systematic reviews, registry studies, and contemporary case series have demonstrated that, in appropriately selected patients, one-stage revision can achieve infection eradication rates comparable to those of two-stage revision while offering important clinical advantages [5,6,7,8]. These advantages include a single surgical procedure, lower perioperative morbidity, shorter hospitalization, earlier rehabilitation, preservation of bone stock, reduced soft-tissue damage, lower healthcare costs, and improved patient satisfaction. Consequently, international consensus statements have progressively expanded the indications for one-stage revision. While earlier recommendations considered factors such as culture-negative infection, resistant microorganisms, or the presence of a sinus tract as contraindications, the most recent International Consensus Meeting (ICM 2025) recognizes that many of these conditions should instead be considered relative rather than absolute contraindications. Current selection criteria primarily emphasize the possibility of performing radical debridement, obtaining adequate soft-tissue coverage, and achieving stable reconstruction in the absence of uncontrolled systemic sepsis [9].

Regardless of the surgical strategy adopted, the successful treatment of PJI depends on several fundamental principles: meticulous radical debridement, the complete removal of infected tissues and implants, stable reconstruction, targeted systemic antimicrobial therapy, and effective local antibiotic delivery. Among these factors, preventing bacterial adhesion to the newly implanted prosthesis has attracted increasing attention. Experimental studies have demonstrated that bacterial colonization begins within minutes after implantation, initiating biofilm formation that subsequently protects microorganisms from both antibiotics and host immune responses. This concept, often referred to as the “race for the surface”, has stimulated the development of technologies capable of protecting implant surfaces during the critical early postoperative period [10].

DAC^®^ (Defensive Antibacterial Coating) hydrogel is a fully resorbable implant coating composed of covalently linked hyaluronic acid and poly-D,L-lactic acid [11]. The hydrogel acts as a temporary physical barrier that reduces early bacterial adhesion while simultaneously serving as a carrier for locally delivered antibiotics selected according to microbiological findings. Complete resorption occurs within approximately 72 h, allowing osseointegration of cementless components without leaving permanent foreign material on the implant’s surface [12]. In vitro studies have consistently demonstrated significant inhibition of bacterial adhesion and biofilm formation, including against methicillin-resistant staphylococci [13]. Experimental animal studies further confirmed the ability of DAC hydrogel to reduce bacterial colonization without impairing bone healing or implant integration [14,15].

Clinical evidence has progressively supported these experimental observations. Several clinical series have reported encouraging infection eradication rates following hip and knee PJI revision surgery when DAC hydrogel was used as an adjunct to meticulous surgical debridement and systemic antibiotic therapy [16,17,18,19,20]. Nevertheless, most available reports include relatively limited patient numbers and, importantly, follow-up durations rarely exceeding three to five years. Consequently, robust evidence regarding the durability of infection eradication and long-term implant survival remains scarce.

Long-term follow-up is particularly relevant in PJI surgery because recurrence may occur several years after revision, and short-term success does not necessarily translate into durable cure. Furthermore, data regarding the use of DAC hydrogel in conjunction with modern hybrid fixation techniques, such as the combination of cemented epiphyseal fixation with uncemented stem, remain limited despite the increasing popularity of this reconstructive strategy.

The purpose of the present study was therefore to evaluate the medium- and long-term clinical, radiographic, and microbiological outcomes of a consecutive series of patients undergoing one-stage revision for infected total knee arthroplasty using the antibiotic-loaded DAC hydrogel. To our knowledge, this represents the longest reported clinical follow-up of one-stage knee revision employing this biodegradable antibacterial coating, providing valuable information regarding its long-term safety, durability, and effectiveness in preventing recurrent infection.

## 2. Results

A total of 52 patients undergoing one-stage revision for peri-prosthetic knee infection (PJI) or for septic knee spacer with the use of DAC antibacterial coating, treated according to the same protocol between 2014 and 2023 were included in the study.

### 2.1. Pre-Operative Data

Age at surgery was 73.2 ± 8.9 years (range 45–87 years), and 32 patients (61.5%) were female. Mean body mass index (BMI) was 29.3 ± 4.4 kg/m^2^ (range: 20.3–40 kg/m^2^) (Table 1).

Forty-eight patients (92.3%) underwent revision for PJI, while four patients (7.7%) were revised for septic spacer in a previously infected knee prosthesis. Concerning clinical presentation, 12 patients (23.1%) showed clinical signs of acute inflammation. No patient had active draining fistulas or soft-tissue defects and implant exposure at the time of our revision surgery. According to the McPherson’s systemic host’s classification [21], 12 patients (23.1%) were classified as type A hosts, 19 (36.5%) as type B, and 21 (40.4%) type C. The most frequent comorbidities included cardiopathy (48.1%), diabetes mellitus or other endocrinopathies (19.2%), respiratory tract disease (13.5%), smoking (13.5%), hepatitis (11.5%), rheumatological disorders (9.6%), chronic renal insufficiency (9.6%), oncological conditions (9.6%), peripheral vasculopathy (7.7%), dermatological (5.8%), and neurological (3.8%) disorders. Anesthesiologic evaluation at the time of surgery graded ten patients ASA 2, 29 as ASA 3, and 13 as ASA 4. Time from first implant was 4.5 ± 4.0 years (range: 0.5–17.0 years). Twenty-three patients (44.2%) had received one or more surgeries to treat the infection prior to our first observation.

### 2.2. Intra-Operative Data

Bone loss was determined intraoperatively, after implant removal, and at the end of surgical debridement. According to the AORI classification [22], 7 (13.5%) patients were classified as AORI type 1, 11 (21.2%) as type 2A, 12 (23.0%) as type 2B and 22 (42.3%) as AORI type 3. Based on bone defect and on capsulo-ligament competence, 15 patients received a hinged prosthesis, while the remaining 37 were treated with a semi-constrained implant.

Surgery duration was 142.5 ± 23.6 min (range: 95–184 min) and blood loss was 460 ± 110 mL (range: 300–650 mL).

### 2.3. Microbiological Data

Microbiological cultures were positive in the majority of cases (Table 2). In four patients (7.7%), no pathogen was found and the diagnosis was confirmed by histology and other parameters according to MSIS 2018 and WAIOT 2020 scores [23,24].

Methicillin-sensitive *Staphylococcus aureus* and *epidermidis* were identified in 7.7% and 11.5% of the cases, respectively. Methicillin-resistant strains of these pathogens were present in 9.6% and 11.5% of the cases, respectively.

Overall, the most commonly isolated organisms were coagulase-negative staphylococci. Less frequent, but still relevant, isolates included Streptococci, *Propionibacterium acnes*, and Enterococci, while mixed flora infections were detected in eight patients (15.4%).

### 2.4. Clinical Outcomes

Two patients died from causes unrelated to the surgical intervention at one and three years, respectively, and two were lost to follow-up at two and three years. Accordingly, 48 patients were clinically evaluable at a follow-up of 7.7 ± 2.9 years (range: 2.5–12.2 years).

The main outcomes were mean increases of 36.7 and 41 points in the KSS score and KSS functional score, respectively, and decreases in pain at rest and at movement from 6.9 and 8.1 prior to surgery to 0.7 and 1.6 at final follow-up, respectively (Table 3).

At final follow-up, 47 of the 48 patients available for clinical evaluation did not show any sign of infection recurrence, resulting in a crude infection-free proportion among final clinically evaluable patients of 97.9%.

**Figure 1:**
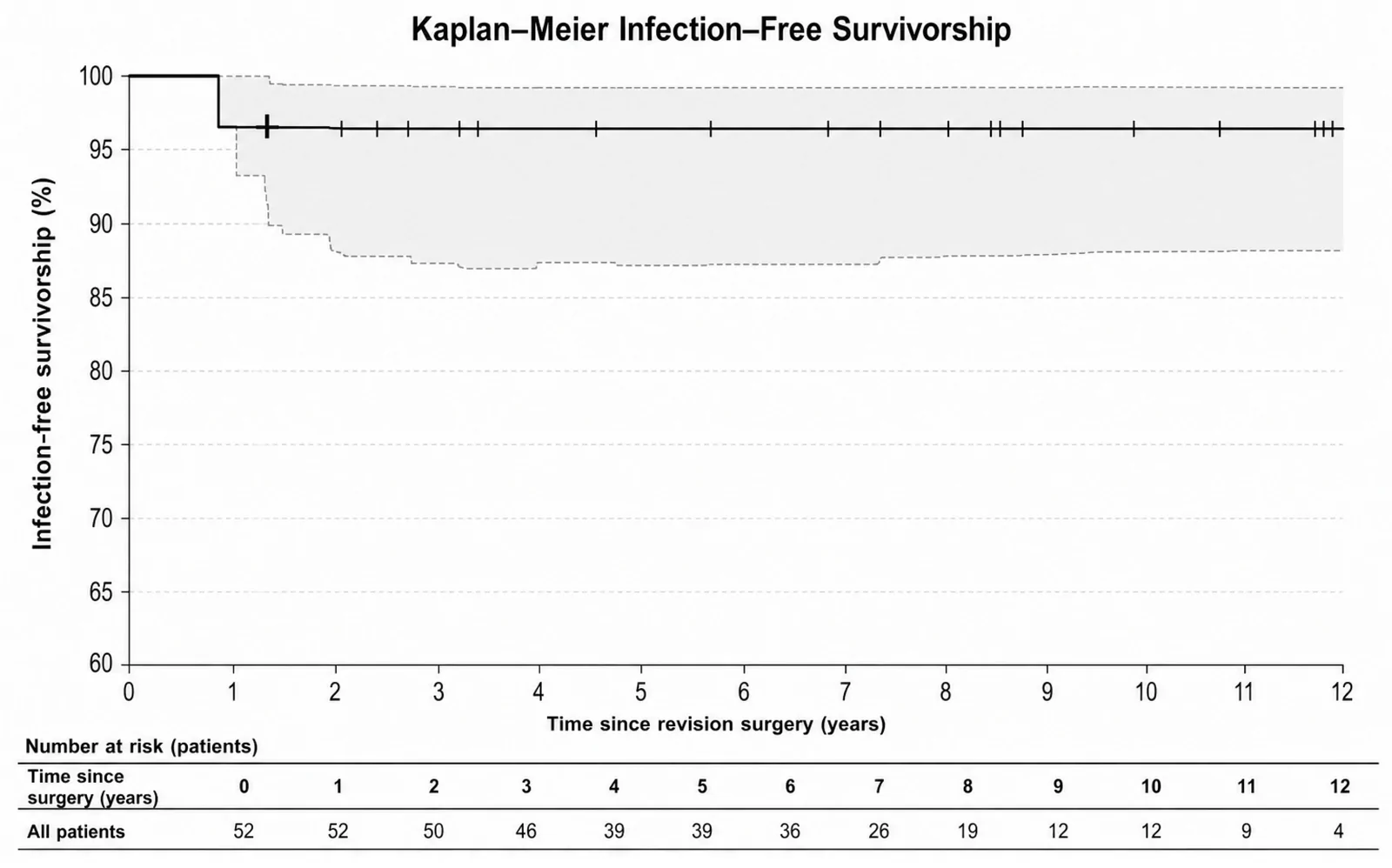
Kaplan–Meier curve showing infection-free survivorship following one-stage revision. Censor marks indicate patients without recurrent infection at their last available follow-up. Shaded/dashed limits represent 95% confidence intervals calculated using the log–log transformation. Kaplan–Meier infection-free survivorship was 98.1% (95% CI, 87.1–99.7%) at 12 years. The number of patients at risk is reported below the graph.

For Kaplan–Meier analysis, all available follow-up information was retained and all of the included 52 patients were appropriately censored at their last known follow-up. On this basis, Kaplan–Meier infection-free survivorship was 98.1% (95% confidence interval (CI), 87.1–99.7%) at 12 years. (Figure 1). The apparent discrepancy with 97.9% reflects two different measures: 47 of the 48 patients available for final clinical evaluation remained infection-free (crude proportion, 97.9%), whereas 98.1% represents the Kaplan–Meier estimate accounting for censoring.

One patient experienced recurrent infection one year after surgery due to a relapse of her recurrent leg erysipelas and a concomitant strongyloidiasis. She underwent above-knee amputation at another hospital.

Laboratory tests showed pre-operative and postoperative values of C-reactive protein (CRP) of 20.9 ± 35.5 mg/L (range: 6–199.4 mg/L) and 5.1 ± 4.2 mg/L (range: 2–11 mg/L), respectively.

Radiographic examination revealed non-progressive lucent lines of <2 mm in 18 patients. Two patients showed radiolucent lines > 2 mm in the tibial component and one patient in both the femoral and tibial component in the absence of clinical signs of loosening.

Concerning complications, two patients required early stiffness treatment by manipulation under anesthesia and one patient suffered deep venous thromboembolism. One patient was treated with internal osteosynthesis in another hospital for peri-prosthetic femoral fracture 6 years after revision. No other patient required additional surgery for infection recurrence or for aseptic loosening.

## 3. Discussion

The main finding of the present study is that one-stage revision combined with DAC antibacterial hydrogel provided excellent long-term infection control and functional outcomes after revision total knee arthroplasty.

At a mean follow-up of 7.7 years, with observations extending to twelve years, 47 of 48 clinically evaluable patients remained infection-free (97.9%). Patients experienced marked improvements in KSS, pain relief, and maintenance of stable implant fixation. To our knowledge, among studies reported in the current peer-reviewed literature on one-stage revision of total knee arthroplasty in which the DAC hydrogel was used, the present study has the longest follow-up.

Long-term evidence is particularly important in the treatment of periprosthetic joint infection because successful short-term eradication does not necessarily translate into durable infection control. The sustained survivorship observed in our series suggests that the local antibacterial protection afforded by DAC hydrogel during the early postoperative period may contribute to preventing bacterial recolonization of newly implanted components while allowing long-term biological fixation to proceed normally. Importantly, no increase in implant-related complications, delayed osseointegration, or mechanical failures attributable to the hydrogel was observed, supporting the long-term safety profile of this biodegradable coating.

Experimental studies have demonstrated that bacterial adhesion to implant surfaces occurs within minutes after implantation, initiating the formation of a mature biofilm that subsequently protects microorganisms from host immune defenses and markedly reduces antibiotic susceptibility. This competition between bacterial colonization and host cell integration, commonly referred to as the “race for the surface”, represents one of the critical determinants of implant-related infection [25,26].

DAC^®^ hydrogel was specifically developed to interfere with this early phase of bacterial adhesion. The biodegradable hyaluronan/poly-D,L-lactide matrix forms a temporary hydrophilic barrier on the implant surface while simultaneously functioning as a carrier for high local concentrations of antibiotics selected according to the microbiological profile. Experimental investigations have consistently demonstrated reduced bacterial adhesion and the inhibition of biofilm formation against both methicillin-sensitive and methicillin-resistant staphylococci, and in vivo studies have confirmed preservation of normal bone healing and implant integration [20].

The excellent long-term results observed in the present series are consistent with this biological mechanism. The absence of aseptic loosening or evidence of impaired biological fixation in our cohort further supports the long-term safety of this technology and suggests that transient local antibacterial protection can be achieved without compromising implant integration.

An additional strength of the present series is the consistent use of hybrid fixation, a solution that provides ease of insertion, possibly improved component alignment, and easy removal if required, compared with fully cemented prostheses [27,28]. In each patient, fixation was achieved with antibiotic-loaded cement limited to the epiphyseal component, whereas the metaphyseal and diaphyseal stems remained uncemented and were coated with antibiotic-loaded DAC hydrogel. This strategy combines the mechanical advantages of biological stem fixation with immediate local antibacterial protection of the uncemented implant surfaces, an aspect that has received little attention in previous reports. The favorable survivorship observed in this cohort supports further investigation of DAC hydrogel in the context of hybrid fixation during one-stage septic revision.

The microbiological spectrum reflected the contemporary epidemiology of knee PJI, with coagulase-negative staphylococci predominating and a substantial proportion of methicillin-resistant organisms [29]. Moreover, we did not observe a different outcome in patients with negative cultural examination, a result that is in line with the recent report from Sabater-Martos et al. [30]. Despite this challenging microbiological profile, infection control remained excellent, even if it is worth noting that the limited number of events in our series prevents conclusions regarding the relative outcome of individual microbiological subgroups. Likewise, almost half of the cohort had undergone previous surgical procedures before referral, and over 40% presented severe bone defects (AORI type 3), highlighting that favorable outcomes were obtained in a complex revision population rather than in highly selected low-risk cases.

The remarkably low loss to follow-up further strengthens the validity of the present findings.

### 3.1. Comparison with Published One-Stage Revision Series

The infection-free survivorship observed in the present study compares favorably with the largest published series of one-stage revision for chronic knee PJI. Historically, one-stage revision has achieved infection eradication rates ranging from approximately 80% to 97%, depending on patient selection, microbiological profile, surgical technique and duration of follow-up [5,6,31,32].

Early European experiences by Freeman et al. [33], Buechel et al. [34] and later by Jenny et al. [35] demonstrated that meticulous debridement combined with immediate reimplantation could achieve durable infection control in carefully selected patients. More contemporary series from high-volume revision centers have reported infection eradication rates generally between 85% and 93%, even in complex revision settings [36,37].

The present study demonstrated that 97.9% of the clinically evaluable patients remained infection-free, despite including a challenging cohort characterized by advanced bone loss, many previous procedures, and a substantial proportion of compromised hosts according to the McPherson classification. Importantly, almost half of the patients had undergone previous surgical treatment for infection before referral, suggesting that our cohort cannot be considered a low-risk population.

Unlike many previous reports in which fully cemented revision constructs were routinely employed [38], all patients in the present series underwent hybrid fixation with cemented epiphyseal fixation and uncemented stems. This reconstructive strategy has become increasingly popular because it facilitates the restoration of alignment and biological fixation while reducing the amount of cement required. However, concerns have occasionally been raised regarding the absence of local antibiotic release around uncemented implant surfaces [39]. The routine application of antibiotic-loaded DAC hydrogel to the uncemented stems may represent an effective solution to this potential limitation, providing temporary local antibacterial protection during the critical period before osseointegration.

### 3.2. Comparison with Systematic Reviews and Meta-Analysis

The present findings are consistent with the conclusions of the most recent systematic reviews and meta-analyses evaluating one-stage revision for knee PJI.

Kunutsor et al. [7], in one of the largest meta-analyses comparing one-stage and two-stage revision, concluded that reinfection rates are broadly comparable between the two procedures when patients are appropriately selected. In a more recent meta-analysis, Xie et al. [8] reported a reinfection rate significantly lower after single-stage revision than after two-stage revision for PJI after knee arthroplasty.

Nagra et al. [40] reviewing exclusively one-stage revision for infected total knee arthroplasty, reported pooled infection eradication rates of approximately 87–89%, although considerable heterogeneity existed among the included studies regarding patient selection, microbiological characteristics, and surgical protocols.

Our results therefore lie at the upper end of the outcomes reported in these pooled analyses. Although direct comparisons should be interpreted cautiously because of differences in inclusion criteria and follow-up duration, our findings support the growing body of evidence indicating that one-stage revision should not be considered an alternative reserved for highly selected patients, but rather a valid standard treatment in experienced multidisciplinary centers.

### 3.3. The Potential Contribution of DAC Antibacterial Hydrogel

Although the retrospective design of the present study does not allow the independent effect of DAC hydrogel to be isolated, several observations deserve consideration.

The antibacterial hydrogel provides temporary local protection during the early postoperative period, when bacterial adhesion and biofilm formation are most likely to occur. This has been recently confirmed in large matched retrospective comparative trials comparing DAC hydrogel coating without the addition of antibiotics both to prevent post-surgical infection after arthroplasty revision [41] and after fracture fixation [42].

The present study substantially expands previous observations [43] by demonstrating that favorable early results are maintained over long-term follow-up.

### 3.4. Study Limitations

The following shortcomings are acknowledged. First, this is a retrospective single-center study including a relatively limited number of patients. Although the cohort is one of the largest homogeneous experiences with DAC hydrogel in one-stage knee revision and follow-up completeness was excellent, larger multicenter investigations would improve external validity. Second, the absence of a control group (DAC not applied to any part of the arthroplasty) prevents direct assessment of the incremental benefit provided by DAC hydrogel. Nevertheless, the observed infection-free survivorship lies at the upper end of the success rates generally reported for one-stage revision in systematic reviews and contemporary series. Third, because all of the procedures followed a standardized multidisciplinary protocol combining meticulous debridement, targeted systemic antibiotics, and local antibacterial coating, the specific contribution of each individual component cannot be isolated. Furthermore, given its retrospective nature, the present study does not allow us to exclude possible patients’ selection bias and to address the possible impact of race and ethnicity, which may affect the generalizability of the study findings.

Overall, these findings support one-stage revision combined with antibiotic-loaded DAC hydrogel as a safe and durable strategy for treating chronic knee PJI. Future prospective multicenter comparative studies should determine whether the addition of DAC hydrogel may have a measurable advantage over conventional one-stage revision and identify patient subgroups that may derive the greatest benefit from this technology.

## 4. Materials and Methods

This retrospective observational study included consecutive patients who underwent one-stage revision total knee arthroplasty for chronic periprosthetic joint infection (PJI) or infected knee spacer at our institution between February 2014 and December 2023. The database was closed on 15 June 2026, which represented the final follow-up date for the present analysis.

Chronic PJI was defined as infection occurring after 4 weeks from the previous surgery or symptoms of greater than 4 weeks’ duration for hematogenous infections [44]. All procedures were performed using a standardized diagnostic, surgical, and postoperative protocol. Exclusion criteria for one-stage revision were refusal of the patient to undergo a one-stage procedure, signs and symptoms of generalized sepsis, or the presence of a draining sinus or soft-tissue defect.

The study was conducted in accordance with the Declaration of Helsinki. All patients provided written informed consent for surgery and for the anonymous use of clinical data for research purposes. According to our institutional policy, the present study did not require an Institutional Review Board approval as all the data were fully de-identified.

The study is reported according to the STROBE statement.

### 4.1. Patient Selection

Patients were eligible if they fulfilled the diagnostic criteria for PJI according to the Musculoskeletal Infection Society (MSIS) 2018 definition [23] and the World Association against Infection in Orthopaedics and Trauma (WAIOT) [24,45] criteria and underwent one-stage revision using DAC^®^ antibacterial hydrogel.

Patients undergoing revision for aseptic failure or incomplete follow-up of less than two years (unless recurrent infection occurred earlier) were excluded.

**Figure 2:**
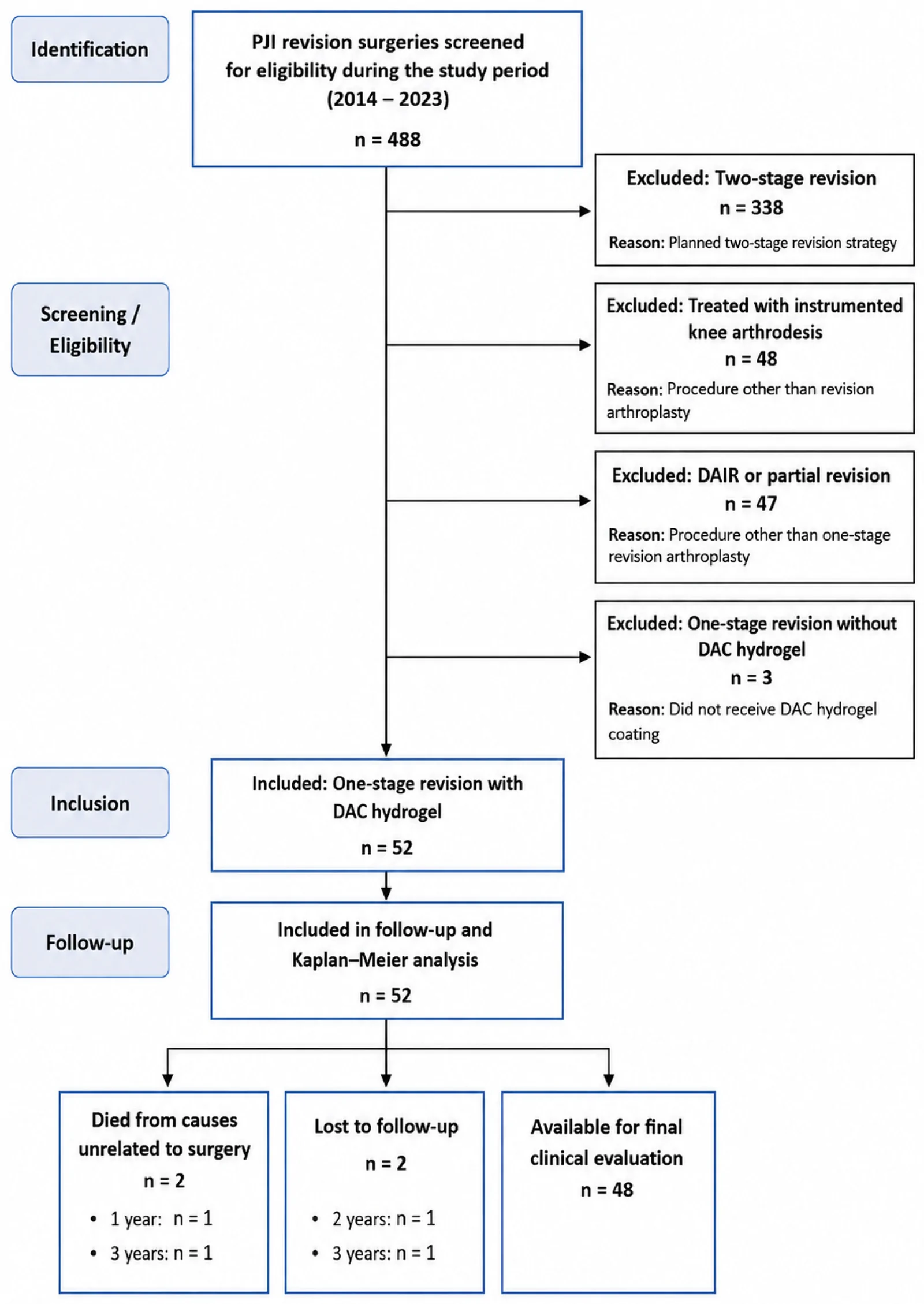
STROBE-style flow diagram of patient screening, inclusion, and follow-up.

Overall, 52 consecutive patients were included, two died from causes unrelated to the surgical intervention at one and three years, respectively, and two were lost to follow-up at two and three years. Accordingly, 48 patients were available for final clinical outcome assessment. (Figure 2).

### 4.2. Pre-Operative Evaluation

All patients underwent a standardized diagnostic work-up including:

- detailed medical history and clinical examination;
- plain radiographs;
- laboratory investigations including CRP, erythrocyte sedimentation rate (ESR), and complete blood count;
- joint aspiration, whenever feasible;
- microbiological cultures; and
- assessment according to the MSIS 2018 [23] and WAIOT diagnostic criteria [24].

General patient condition was classified according to:

- American Society of Anesthesiologists (ASA) score [46];
- McPherson host classification [21]; and
- Anderson Orthopaedic Research Institute (AORI) classification for bone loss (assessed intraoperatively after implant removal) [22].

### 4.3. Surgical Technique

All procedures were performed through the previous surgical incision whenever possible.

Following removal of all prosthetic components and cement, extensive radical debridement was performed, including complete excision of infected synovium, scar tissue, necrotic soft tissues, and devitalized bone. A large number of tissue specimens (minimum five) were collected from different anatomical sites for microbiological and histological examination.

Bone defects were reconstructed according to the AORI classification using a combination of modular augments, metaphyseal cones, antibiotic-loaded bone cement, and long stems, whenever required. Structural allografts were not used.

Depending on ligament competence and residual bone stock, patients received either:

- a rotating-hinge revision prosthesis (LINK Endo-Model^®^, Waldemar Link, Hamburg, Germany), or
- a semi-constrained TC3 prosthesis (DePuy Synthes^®^, Warsaw, IN, USA).

Hybrid fixation was adopted in all cases. Epiphyseal fixation was achieved using commercially available antibiotic-loaded polymethylmethacrylate cement containing gentamicin combined with vancomycin (COPAL^®^ G+C/V, Heraeus Medical GmbH, Wehrheim, Germany)or VancoGenX^®^ (Tecres S.p.A., Sommacampagna (VR), Italy),according to availability), whereas metaphyseal and diaphyseal stems were implanted without cement.

Bone defects were managed using antibiotic-loaded cement, augments, metaphyseal cones, and long stems according to defect severity. No allografts were used in any case.

Tourniquet was only applied during cementation. No drains were used.

### 4.4. DAC Antibacterial Hydrogel

Immediately before implantation, all prosthetic components were coated with a sterile biodegradable hydrogel (DAC^®^, Novagenit S.r.l., Mezzolombardo, Italy).

**Figure 3:**
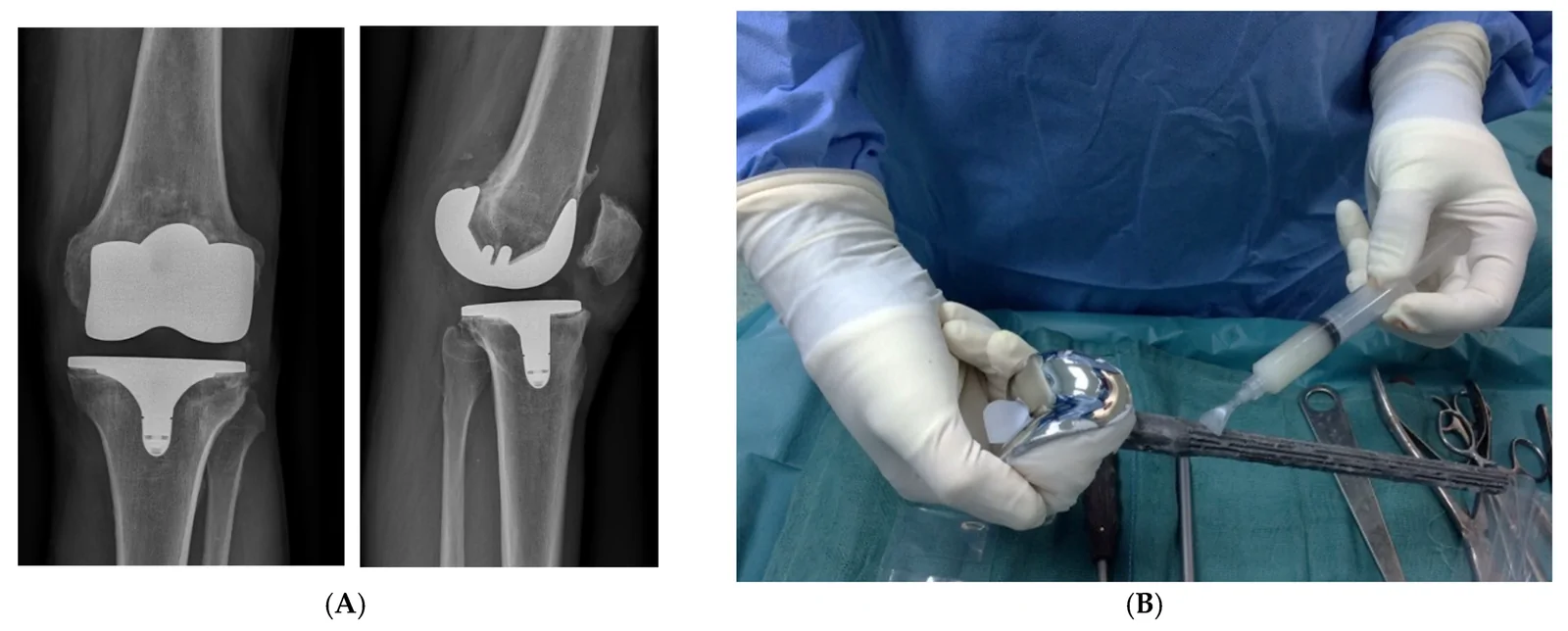

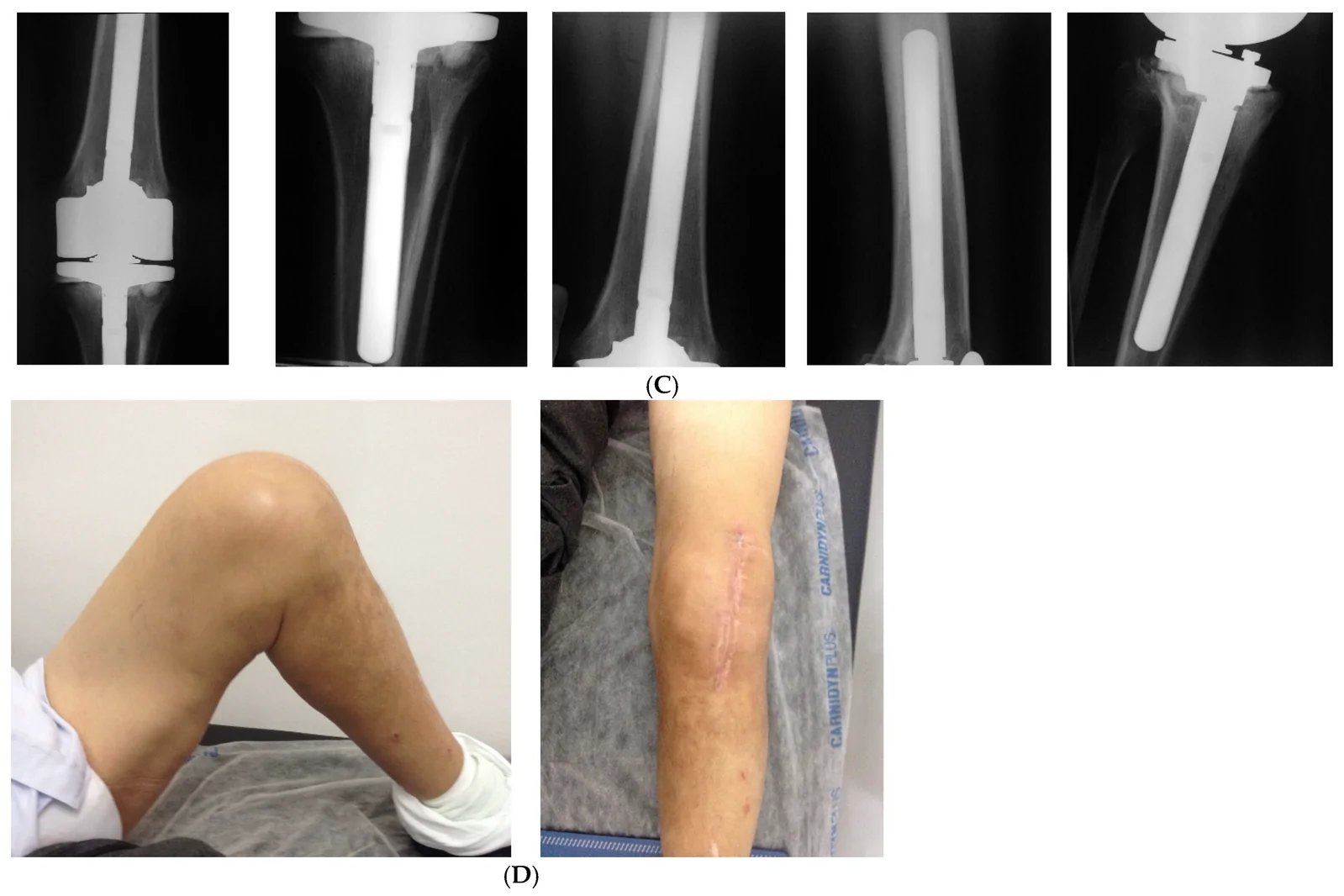
Patient #13. Clinical example of the DAC antibiotic-loaded hydrogel coating application. (**A**) Pre-operative radiographic examination. Ligament instability. MRSA positive culture. (**B**) Intra-operative picture, showing DAC hydrogel application on the femoral implant, prior to its insertion. (**C**) Radiographic examination 6 years after surgery. (**D**) Clinical outcome.

In each of the first ten patients, the DAC^®^ hydrogel coating was loaded with either 5 wt./wt.% vancomycin or 5 wt./wt.% vancomycin + 5 wt./wt.% meropenem, on the basis of the results of examination of pathogen isolates. In each of the remaining 42 patients and in all cases with negative culture examination, 5 wt./wt.% vancomycin + 5 wt./wt.% meropenem was used. In each patient, 10–15 mL of the coating was applied to the uncemented intra-medullary parts of the implant, the polyethylene insert, and the extra-medullary surface (cf. Figure 3).

### 4.5. Postoperative Management

Systemic antibiotic treatment was administered for 31.8 ± 6.3 days (range: 24–48 days). Each patient received dual antibiotic treatment, based on antibiogram results. During the hospital stay and until antibiogram results, a combination of glycopeptide and tazobactam-piperacillin or meropenem were administered. At discharge and whenever possible, antibiotic treatment was shifted to oral administration.

Besides antibiotic treatment, each patient received thromboprophylaxis for 5 weeks with low-dose heparin.

Postoperative rehabilitation was initiated on the first postoperative day with immediate mobilization and progressive weight bearing according to implant stability and soft-tissue conditions.

### 4.6. Follow-Up Laboratory, and Radiographic Examination

Patients were reviewed every 3–6 months during the first postoperative year and annually thereafter.

Clinical assessment included:

- Knee Society Score (KSS) [47];
- Knee Society Functional Score;
- Visual Analogue Scale (VAS) for pain at rest and during movement.

Laboratory follow-up included serum CRP.

Standardized anteroposterior and lateral radiographs were obtained at each follow-up visit. Radiographic evaluation assessed component position, radiolucent lines, and evidence of aseptic loosening.

The primary endpoint was infection eradication, defined as the absence of clinical, laboratory, and radiographic evidence of recurrent infection and no need for additional surgery for infection recurrence.

Secondary endpoints included:

- implant survivorship;
- functional outcome;
- radiographic stability;
- postoperative complications; and
- need for further revision surgery.

### 4.7. Statistical Analysis

Continuous variables are reported as mean ± standard deviation (SD) with ranges. Categorical variables are expressed as frequencies and percentages.

Changes in clinical outcome scores between pre-operative assessment and final follow-up were analyzed using paired Student’s *t*-tests after confirmation of normal distribution. Statistical significance was set at *p* < 0.05.

Infection-free implant survivorship was estimated using the Kaplan–Meier method, with recurrent infection considered the endpoint and patients without recurrence censored at their latest follow-up.

Statistical analyses were performed using SPSS version 27 (IBM Corp., Armonk, NY, USA).

## Figures and Tables

**Table 1:** Pre-operative data. Abbreviations: BMI: Body Mass Index. SD: Standard Deviation.

#	Date of Surgery	Age (yr)	F	M	BMI (kg/m^2^)	Diagnosis		Host’s Type		
						PJI	Septic spacer	A	B	C
1	13/2/2014	73	1		32.1	1				1
2	13/2/2014	83	1		34.5	1				1
3	24/2/2014	75		1	38.3		1			1
4	10/3/2014	72	1		29.1	1				1
5	17/4/2014	55		1	35		1			1
6	28/4/2014	86	1		35.2	1				1
7	14/7/2014	55	1		33.7	1				1
8	22/9/2014	78	1		34.1		1			1
9	9/10/2014	72		1	29	1				1
10	12/1/2015	73	1		30.3		1	1		
11	9/2/2015	65	1		36	1			1	
12	23/3/2015	66	1		33.2	1		1		
13	26/3/2015	82	1		25.9	1		1		
14	15/6/2015	62	1		31.3	1				1
15	18/6/2015	70		1	34.2	1				1
16	11/1/2018	83	1		37.1	1			1	
17	15/2/2018	79	1		29.2	1				1
18	15/2/2018	79	1		25.9	1		1		
19	6/3/2018	76	1		20.3	1		1		
20	22/3/2018	81		1	25.5	1			1	
21	5/4/2018	72	1		36.7	1			1	
22	26/4/2018	78		1	26.8	1				1
23	3/5/2018	75	1		31.2	1			1	
24	14/6/2018	78	1		24.6	1		1		
25	13/9/2018	75	1		27.1	1			1	
26	14/2/2019	82		1	25.3	1		1		
27	28/2/2019	71	1		23.7	1				1
28	7/3/2019	79	1		23	1				1
29	11/4/2019	82	1		29.4	1		1		
30	9/5/2019	83	1		25.8	1			1	
31	24/5/2019	57		1	27.7	1			1	
32	4/6/2019	69	1		33.3	1				1
33	2/7/2019	63		1	24.9	1		1		
34	3/9/2019	87	1		29.4	1				1
35	5/9/2019	73	1		26.1	1			1	
36	26/9/2019	82		1	30.8	1				1
37	5/112019	69	1		24	1				1
38	4/2/2020	45		1	26.3	1			1	
39	10/3/2020	81	1		25.9	1			1	
40	2/4/2020	71	1		30.8	1			1	
41	18/6/2020	73		1	25.6	1			1	
42	13/10/2020	77		1	29.4	1			1	
43	18/2/2021	83	1		24.6	1				1
44	8/4/2021	69		1	40	1			1	
45	15/6/2022	85		1	22.4	1			1	
46	7/9/2022	58		1	28	1		1		
47	2/11/2022	70	1		25.8	1			1	
48	8/3/2023	81	1		30	1				1
49	24/5/2023	67		1	29.2	1			1	
50	6/9/2023	76		1	31	1			1	
51	4/10/2023	65		1	26.4	1		1		
52	12/12/2023	67		1	28	1		1		
TOTAL			32	20		48	4	12	19	21
%			61.5	38.5		92.3	7.7	23.1	36.5	40.4
Mean		73.2			29.3					
SD		8.9			4.4					
Min		45			20.3					
Max		87			40.0					

**Table 2:** Cultural examination results.

Pathogen	Number of Isolates	%
Methicillin-Sensitive *Staphylococcus aureus* (MSSA)	4	7.7
Methicillin-Sensitive *Staphylococcus epidermidis* (MSSE)	6	11.5
Methicillin-Resistant *Staphylococcus aureus* (MRSA)	5	9.6
Methicillin-Resistant *Staphylococcus epidermidis* (MRSE)	6	11.5
*Staphylococcus lugdunensis*	4	7.7
Other Coagulase-Negative *Staphylococci*	7	13.5
*Streptococci* spp.	4	7.7
*Cutibacterium acnes*	4	7.7
*Enterococci* spp.	3	5.8
*Serratia* spp.	1	1.9
*Escherichia coli*	1	1.9
Others	5	9.6

**Table 3:** Clinical outcomes. Abbreviations: KSS: Knee Society Score. VAS: Visual Analogue Score.

	Pre-Operative	At Final Follow-Up	*p*
KSS Score	47.7 ± 13.5 (range 22–68)	84.4 ± 12.5 (range 60–88)	<0.0001
KSS Score Function	41.9 ± 9.0 (range 18–59)	82.9 ± 10.1 (range 58–88)	<0.0001
Pain at Rest (VAS Score)	6.9 ± 3.6 (range 5–10)	0.7 ± 2.0 (range 0–2)	<0.0001
Pain at Movement (VAS Score)	8.1 ± 3.8 (range 6–10)	1.6 ± 2.7 (range 0–3)	<0.0001

## Data Availability

The data presented in this study are available on request from the corresponding author due to privacy restrictions.
